# Automated Signal Quality Assessment for Heart Sound Signal by Novel Features and Evaluation in Open Public Datasets

**DOI:** 10.1155/2021/7565398

**Published:** 2021-02-24

**Authors:** Hong Tang, Miao Wang, Yating Hu, Binbin Guo, Ting Li

**Affiliations:** ^1^School of Biomedical Engineering, Dalian University of Technology, Dalian 116024, China; ^2^Liaoning Key Lab of Integrated Circuit and Biomedical Electronic System, China; ^3^College of Information and Communication Engineering, Dalian Minzu University, Dalian, China

## Abstract

Automated heart sound signal quality assessment is a necessary step for reliable analysis of heart sound signal. An unavoidable processing step for this objective is the heart sound segmentation, which is still a challenging task from a technical viewpoint. In this study, ten features are defined to evaluate the quality of heart sound signal without segmentation. The ten features come from kurtosis, energy ratio, frequency-smoothed envelope, and degree of sound periodicity, where five of them are novel in signal quality assessment. We have collected a total of 7893 recordings from open public heart sound databases and performed manual annotation for each recording as gold standard quality label. The signal quality is classified based on two schemes: binary classification (“unacceptable” and “acceptable”) and triple classification (“unacceptable”, “good,” and “excellent”). Sequential forward feature selection shows that the feature “the degree of periodicity” gives an accuracy rate of 73.1% in binary SVM classification. The top five features dominate the classification performance and give an accuracy rate of 92%. The binary classifier has excellent generalization ability since the accuracy rate reaches to (90.4 ± 0.5) % even if 10% of the data is used to train the classifier. The rate increases to (94.3 ± 0.7) % in 10-fold validation. The triple classification has an accuracy rate of (85.7 ± 0.6) % in 10-fold validation. The results verify the effectiveness of the signal quality assessment, which could serve as a potential candidate as a preprocessing in future automatic heart sound analysis in clinical application.

## 1. Background

Heart sounds are acoustic vibrations generated due to the beating of the heart and blood flow therein. Specifically, the sounds reflect the hemodynamic changes associated with heart valves snapping shut [1, 2]. There is a natural link exits between the heart sound and the condition of the heart, and it was established after the invention of the stethoscope by Rene Laennec in 1816. Physicians usually prefer cardiac auscultation to diagnose cardiovascular diseases [3]. Computer-aided algorithms are necessary to avoid the limitations of the human listening system and manual work in screening cardiovascular diseases using digital heart sound signal. A recent review on this topic showed that more than 1,300 research articles are available from 1963 to 2018 [4, 5]. Although a lot of research work has been done on segmentation, feature extraction, and classification, it is still an open area for researchers to develop automatic and robust algorithms for the identification and classification of various events in cardiac sound signals. The key problem associated with this approach is the recording of less informative heart sounds by an unskilled people. The quality of heart sound signal has an obvious impact on the output of the automatic diagnostic system. Hence, we need a high quality heart sound signal to avoid misinterpretation of heart diseases and for more accurate classification of heart sounds.

There are generally two ways available to obtain high quality signals: hardware- and software-based protocols. In the first approach, a very sensitive sensor is designed to detect heart sound for better identification of turbulent blood flow (e.g., a very light-weight, dual accelerometer has been developed by Semmlow to collect high quality heart sound on the chest surface) [6]. Recently, Roy et al. aimed to design an electronic stethoscope which would assist doctors to analyze the heart sound and identify a disease condition of the heart [7]. On the other hand, the software-based approach estimates the signal quality and selects high quality components for further processing based on computer analysis. Previous researchers have proposed some methods for quality assessment of heart sound signals. Beritelli et al. proposed a selection algorithm in 2009 to determine the best subsequence from a signal based on cepstral distance measurement [8]. Another best subsequence selection algorithm was proposed by Li et al. based on the degree of heart sound periodicity [9–12]. Abdollahpur et al. proposed a cycle quality assessment method to select those cycles with little noise or spikes [13]. The first binary signal quality classification algorithm was proposed by Nazeri et al. using energy-based and noise level-based quality measurement [14] in 2012. Zabihi et al. detected abnormalities, and quality used 40 features extracted from linear predictive coefficients, entropy, mel frequency, cepstral coefficient, discrete wavelet coefficients, and power spectral density [15]. An ensemble neural network was trained and tested for binary quality classification. Springer et al. proposed an excellent algorithm using nine features, and a linear discrimination classification was used to perform binary classification [16, 17]. Mubarak et al. proposed the latest algorithm in 2018, where three features in the time domain were used to assess signal quality [18].

Previous algorithms [16–18] considered the segmentation of heart sounds as a preprocessing step. Therefore, the performance of quality assessment technique would depend on the accuracy of segmentation. On the other hand, the segmentation operation also increases the computational complexity of the algorithm. A common problem associated with these existing algorithms is that they were seldom validated widely in various environments. They were usually validated solely by recordings collected by one type of heart sound sensor or recordings collected in one scenario.

This study is aimed at extracting effective features for automatic signal quality assessment. The authors assume that the signal quality can be reflected by kurtosis, energy ratio in frequency bands, signal envelope, envelope of signal autocorrelation, and sound periodicity. The features could have different contributions for quality assessment. Furthermore, signal quality could be classified by an SVM network based on these features.

## 2. Methods

### 2.1. Dataset

In this study, data for signal quality assessment were collected from four sources. They are listed in the following.

- Physionet/CinC Challenge (CinCHS) 2016 HS Database [19, 20]: these recordings were collected from various positions on the chest surface at different environments including home, hospital, and uncontrolled surroundings. It consists of 3153 recordings collected from 765 subjects. The detail description is given in [19].
- Pascal Classifying Heart Sound Challenge (PASCAL) Database [21]: the data were collected from two sources. One was from an iPhone app, and another was from a clinic trial in a hospital using a digital stethoscope. There are 859 recordings available.
- Heart Sounds Catania (CTHS) Database 2011 [22, 23]: this database was a collection of heart sounds used for biometry by the University of Catania, Italy. It contained heart sounds acquired from 206 people using a digital stethoscope. There are 412 recordings available. The data can be downloaded at [22].
- Cardiac disease heart sound (CDHS) Database: It included 3875 recordings acquired by the authors' group from 76 patients in the second attached hospital of Dalian Medical University since 2015.

The sampling frequencies in the four datasets are different. They are 2000 Hz, 11025 Hz, 44100 Hz, and 2000 Hz in CinCHS, CTHS, PASCAL, and CDHS, respectively. The four databases provide 8299 recordings available. However, to ensure that the signal quality can be reliably assessed, those recordings with time length less than 6 s are excluded. It is found that the noise to cause low signal quality is mainly respiratory sounds, environment noise, and skin contact.

### 2.2. Signal Annotations

To develop an automatic signal quality classification algorithm, gold standard annotations for the signal quality of each recording are needed. These gold annotations were done by one skilled physician and two senior researchers with 10 years of experience in the field of heart sound signal processing. Each annotator did these annotations in quiet environments using both headphone listening and visual examination. Each recording was assigned a quality label rating of “1” to “5” according to the label scheme given in Table 1.

**Figure 1:**
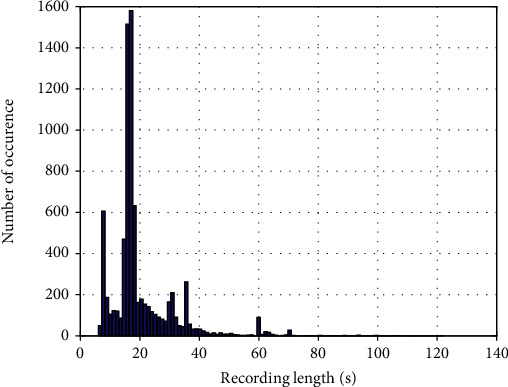
Histogram statistics of recording length.

**Figure 2:**
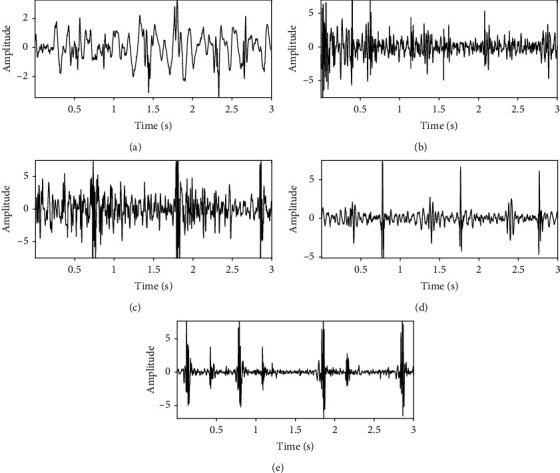
Typical examples of different qualities of heart sound signals: (a) very bad quality, (b) bad quality, (c) borderline quality, (d) good quality, and (e) excellent quality.

It is necessary to combine the annotations into a single annotation for each recording. The round-off operation to the average of the annotations produces the final label. The number of annotated recordings is summarized in Table 2. The distribution of signal length is analyzed by histogram and shown in Figure 1. Most of the recordings have a time length around 16 s. Finally, 7893 recordings are remained for signal quality assessment. It shows that 319 recordings are “very bad,” 2187 recordings are “bad,” 1880 recordings are borderline quality, 1950 recordings are “good,” and 1557 recordings are “excellent.” The typical examples of “very band,” “bad,” “borderline,” “good,” and “excellent” are illustrated in Figure 2. It can be seen from these figures that high-quality signals exhibit large amplitude and cyclic in nature. However, low-quality signals show heavy random noise or spikes. The heart sound data and labels are open for free public access at Baidu Netdisk.

### 2.3. Framework of the Proposed Algorithm

**Figure 3:**
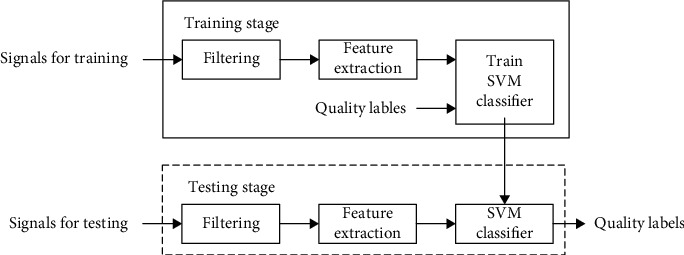
Flow chart of the proposed signal quality assessment.

Figure 3 shows the work flow of the supervised classification scheme. Signals are separated into two subsets. One is for training and the other is for testing. In the training stage, each signal passes through an antialiasing filter and then is down sampled to 1000 Hz. Baseline wandering is removed by a high-pass 3-order Butterworth filter with cut-off frequency of 2 Hz. After that, all heart sound signals were preprocessed to be zero mean and standard deviation before any further analysis. Then, quality labels and features are used to train the SVM classifier. In the testing stage, features are extracted as the same as those in the testing stage and input to the classifier to get quality prediction labels.

### 2.4. Feature Extraction

#### 2.4.1. Features Related to Heart Sound Signal

*(1) Kurtosis of Heart Sound Signal*. Suppose that *x*(*n*) is a real digital sequence of a heart sound recording after preprocessing. It has zero average and standard deviation. The kurtosis is defined as

(1) $$\begin{array}{c} K_{x}=E\left[{\left(x\right)}^{4}\right]/{\left(E\left[{\left(x\right)}^{2}\right]\right)}^{2}, \end{array}$$

where *E*(·) is the expectation operator. Kurtosis is a fourth-order statistical moment and used to measure how much a random process is close to a Gaussian process [24–27]. If a random process is Gaussian, the kurtosis is equal to 3. The kurtosis is less than 3 for a sub-Gaussian process and greater than 3 for super-Gaussian process. A basic acceptable assumption for heart sound signals and noise is that the noise is always stochastic; however, heart sounds and murmurs (if any) are somewhat periodic. The noise is somewhat like a Gaussian process because of the central limit theorem. Hence, a heart sound signal with little noise is expected to have prominent heart sounds or murmurs. The kurtosis of a low-noise heart sound signal would have a large value in high possibility.

*(2) Energy Ratio of Low Frequency Band*. Previous studies show that the dominant frequencies of the first and second heart sounds are generally greater than 24 Hz and less than 144 Hz [16, 17]. The random noise in heart sound signal may have a wide frequency band. The comparison of energy in the spectral band of heart sound signal and total energy may provide a measure of noise, and hence equally, a measure of signal quality. The energy ratio of the low frequency band is defined as

(2) $$\begin{array}{c} r_{\text{e}\_\text{low}}=\overset{144}{\underset{f=24}{\sum }}P_{x}\left(f\right)/\overset{500}{\underset{f=0}{\sum }}P_{x}\left(f\right), \end{array}$$

where *P*_*x*_(*f*) is the power spectral density of the heart sound signal. It is estimated using Welch's method where the signal is divided into the longest possible sections, to get as close as to but not exceeding 8 segments with 50% overlap. A modified periodogram is computed for each segment using Hamming window, and all the resulting periodograms are averaged to compute the final spectral estimate. This ratio is expected to be positively correlated to signal quality.

*(3) Energy Ratio of High Frequency Band*. This feature is defined similarly as that in (2) except that the frequency range considered is [200 500] Hz. Based on the analysis mentioned above, the signal associated with this frequency band is possibly related to noise or murmurs.

*(4) Energy Ratio of Middle Frequency Band*. It is calculated by the energy scale in the middle frequency band within [144 200] Hz.

#### 2.4.2. Features Related to the New Frequency-Smoothed Envelope

A heart sound signal is complex and highly nonstationary in nature. The envelope would give passable information in investigating of repeating patterns in noisy environments. Previous researchers have proposed several envelope algorithms [28–31]. The first envelope algorithm may be the Shannon envelope calculated from Shannon energy by Liang et al. in 1997 for heart sound segmentation [29]. Hilbert envelope was obtained via moving average of the analytical signal. Choi et al. proposed a characteristic waveform where the envelope was defined as the output of a single-degree-of-freedom model [30]. Gupta et al. carried out their study based on envelope calculated from Shannon energy using a continuous time window of 0.02 s with 0.01 s overlap [31].

It can be seen that the existing envelope algorithms employ moving average filtering operation in the time domain to remove high frequency components. In this study, a new frequency-smoothed envelope is proposed. Consequently, novel features can be defined.

**Figure 4:**
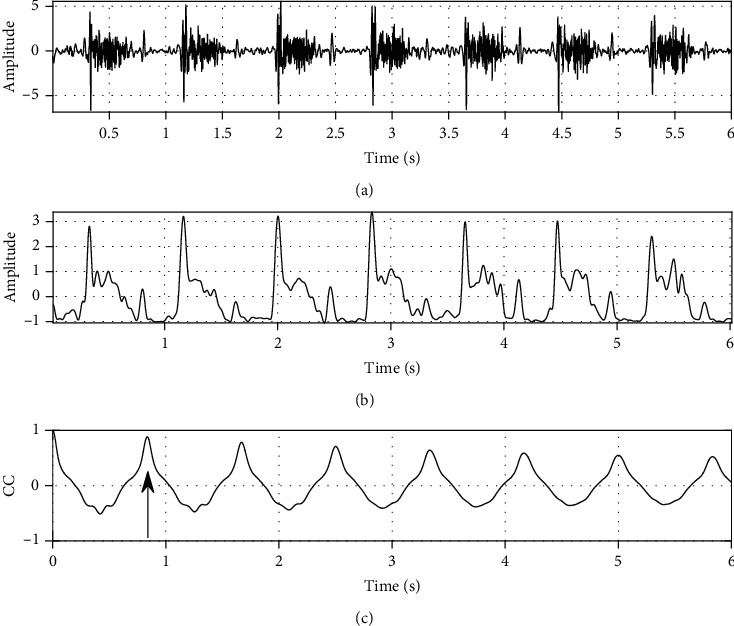
A typical example of frequency-smoothed envelope with excellent quality: (a) a heart sound signal with excellent quality, (b) frequency-smoothed envelope, and (c) normalized autocorrelation function of the envelope.

Discrete short-time Fourier transform (STFT) is applied to a heart sound digital sequence, *x*(*n*),

(3) $$\begin{array}{c} {STFT}_{x}\left(m,k\right)=\overset{\infty }{\underset{n=-\infty }{\sum }}x\left(n\right)w\left(n-m\right){\text{e}}^{-j2\pi \left(kF\right)\left(n/f_{s}\right)}, \end{array}$$

where *w*(*n*) is a sliding window, *F* is the sampling interval in frequency domain, and *f*_*s*_ is the sampling frequency in the time domain. Integral operation to the magnitude of *STFT*_*x*_(*m*, *k*) with respect to the frequency domain produces an envelope of the heart sound signal and defined as

(4) $$\begin{array}{c} e\left(m\right)=\frac{1}{K}\overset{\infty }{\underset{k=-\infty }{\sum }}\left|{STFT}_{x}\left(m,k\right)\right|, \end{array}$$

where *K* is the number of frequency bins. It is seen that the average filtering is operated in the frequency domain. The envelope is therefore called frequency-smoothed envelope and shown in Figure 4. It is found that the envelope matches the signal very well. In this study, the time-domain sampling frequency of a digital heart sound signal is 1000 Hz, the sliding window is a rectangle with time width 0.03 s (30 samples), and the overlapping samples is 29.

*(1) Standard Deviation of the Envelope*. Standard deviation indicates how much the degree of sample is away from the mean in a distribution. Hence, the envelope of a noise-free signal could have greater standard deviation than that with noise.

*(2) Sample Entropy of the Envelope*. The sample entropy is a measure of the complexity of a signal [32]. It can be seen that the envelope is highly periodic for a high quality heart sound signal. The sample entropy should be low value due to this regularity. On the contrary, the sample entropy should increase with the envelope of a noisy signal. The algorithm to calculate sample entropy can be found in [32]. To reduce the computation load, the envelope is down sampled to 30 Hz.

#### 2.4.3. Features Related to Autocorrelation of the Envelope

The normalized autocorrelation function of the envelope is

(5) $$\begin{array}{c} r\left(l\right)=\overset{\infty }{\underset{m=-\infty }{\sum }}e\left(m\right)e\left(m-l\right)/\overset{\infty }{\underset{m=-\infty }{\sum }}e^{2}\left(m\right), \end{array}$$

where *l* is the time delay. It is known from the mechanism of heart sound generation that the heart sound events and murmurs are quasiperiodic. The quasiperiodicity can be clearly reflected by the envelope. The autocorrelation function emphasizes the repeating patterns of these sounds and murmurs [33]. As can be seen in Figure 4(c), a dominant peak, indicated by the arrow, occurs at a time delay due to the high correlation between sounds in adjacent cycles. It could be safely concluded that low peaks would be seen with a bad quality signal.

*(1) Maximum Peak in the Normalized Autocorrelation Function of the Envelope between Delay Times of 0.3 S to 2.5 S*. The maximum peak between 0.3 s and 2.5 s is used, as indicated by an arrow in Figure 4(c), and the noise signal contains a higher magnitude peak in the specified range. In this reasoning, the peak value is able to reflect the signal quality in some degree. The delay time generally corresponds to the cardiac period. A very wide range of the cardiac periods is considered in this study. The minimum cycle period in consideration is 0.3 s corresponding to 200 beats per minute, and the maximum cycle period is 2.0 s corresponding to 30 beats per minute [34]. Formula (6) was the feature of the maximum peak in the normalized autocorrelation function of the envelope between 0.3 s and 2.0 s. The authors got this feature by searching the maximum of *r*(*l*) (the normalized autocorrelation function of the envelope) where the time delay *l* is between 0.3∗*f*_*s*_ and 2∗*f*_*s*_, where *f*_*s*_ = 1000. This feature reflects the degree of correlation between sounds in adjacent cycles. This feature is defined as

(6) $$\begin{array}{c} A_{r}=\max\left[r\left(l\right)\right],\;0.3*f_{s}\leq l\leq 2*f_{s}, \end{array}$$

where max(·) is to get the maximum magnitude. For the reason to reduce the amount of data to be analyzed, the first 6 s of autocorrelation is used in this study.

*(2) Kurtosis of the Normalized Autocorrelation Function*. In the authors' reasoning, the autocorrelation function of a high quality signal would be far away from the Gaussian distribution. Hence, the kurtosis of the autocorrelation function could have a high value. The calculation for this kurtosis is given in (1).

*(3) Sample Entropy of the Normalized Autocorrelation Function*. Similarly, the autocorrelation function of a high quality signal is expected to have high regularity. Thus, the sample entropy could have a low value. The algorithm calculates the sample entropy that can be found in [32]. To reduce the computation load, the autocorrelation function is down sampled to 30 Hz.

#### 2.4.4. Features Extracted from the Cycle Frequency Domain

A heart sound signal is safely believed to be quasiperiodic [9–11, 35], and an indicator to evaluate quantitively the degree of periodicity has been proposed in [9–11] in the cycle frequency domain. If the cycle duration of a heart sound signal is *T*, the time-varying autocorrelation is

(7) $$\begin{array}{c} R_{x}\left(t,\tau \right)\overset{\Delta }{=}\underset{N\to \infty }{\lim}\frac{1}{2N+1}\overset{N}{\underset{n=-N}{\sum }}x\left(t+\tau /2+nT\right)x^{*}\left(t-\tau /2+nT\right). \end{array}$$

*R*_*x*_(*t*, *τ*) is a periodic function. *N* is the number of cycles involved in analysis. *R*_*x*_(*t*, *τ*) can be rewritten using the Fourier series as

(8) $$\begin{array}{c} R_{x}\left(t,\tau \right)=\overset{+\infty }{\underset{\alpha =-\infty }{\sum }}R_{x}\left(\alpha ,\tau \right)e^{j\;2\pi \;\alpha \;t}, \end{array}$$

where *α* is a real number. It is called the cycle frequency.

The coefficient of the Fourier series is

(9) $$\begin{array}{c} R_{x}\left(\alpha ,\tau \right)={\langle x\left(t+\tau /2\right)x^{*}\left(t-\tau /2\right)e^{-j2\pi \;\alpha \;t}\rangle }_{t}, \end{array}$$

where the operator <·>_*t*_ denotes the time average. *R*_*x*_(*α*, *τ*) is called the cyclic correlation function. It degenerates into a traditional correlation when the cycle frequency *α* becomes zero. In the extreme case, the basic cycle frequency of the heart sound signal is *α*=1/*T*. *R*_*x*_(*α*, *τ*)≠0 only if cycle frequency is *kα* and *R*_*x*_(*α*, *τ*)=0 elsewhere, where *k* is an integer. However, the cycle duration of a normal heart sound signal is not fixed, and it varies with time. This is known as heart rate variability. Thus, in practice, *R*_*x*_(*α*, *τ*)≠0 if *α* is any real number. *R*_*x*_(*α*, *τ*) can be transformed into the frequency domain via the Fourier transform. That is,

(10) $$\begin{array}{c} S_{x}\left(\alpha ,f\right)=\int_{-\infty }^{\infty }R_{x}\left(\alpha ,\tau \right)e^{-j2\pi \;f\tau }\text{d}\tau . \end{array}$$

*S*_*x*_(*α*, *f*) is called the cyclic spectral density. In any stochastic process for which *R*_*x*_(*α*, *τ*)≠0 or *S*_*x*_(*α*, *f*)≠0, the process exhibits a certain degree of periodicity at cycle frequency *α*. The analysis in the cycle frequency domain is of primary interest. An integral is operated over the frequency domain to get the cycle frequency spectral density (CFSD)

(11) $$\begin{array}{c} \gamma_{x}\left(\alpha \right)=\int_{-\infty }^{\infty }\;|\;S_{x}^{\alpha }\left(f\right)\;|\;\text{d}f. \end{array}$$

**Figure 5:**
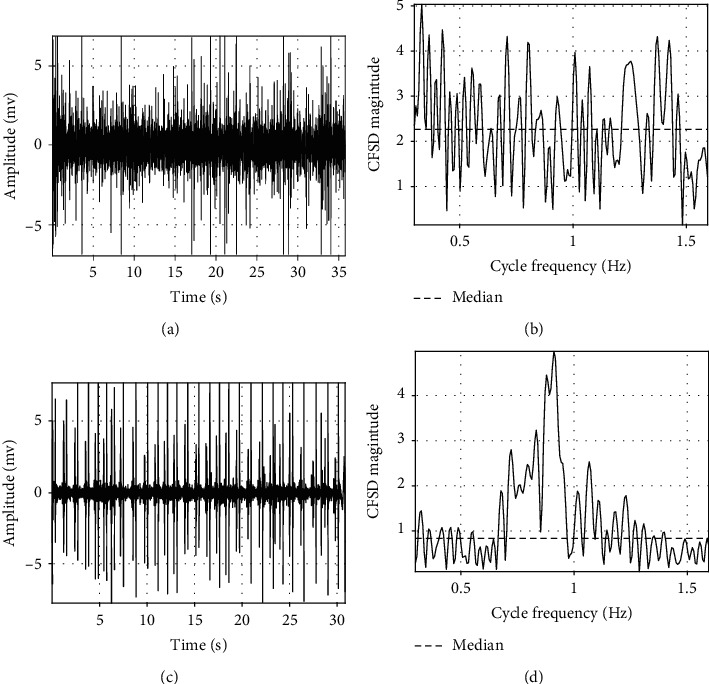
An example to extract the degree of periodicity. (a) An “unacceptable” signal, (b) CFSD of the “unacceptable” signal; there is no dominant peak. (c) An “acceptable” signal. (d) CFSD of the “acceptable” signal. The dominant peak has a much higher magnitude than the median.

*(1) Degree of Sound Periodicity*. A quality indicator is then defined to reflect the degree of sound periodicity. It is somewhat equal to consider the dominant peak of CFSD

(12) $$\begin{array}{c} {dp}_{x}=\frac{\max\left(\gamma_{x}\left(\alpha \right)\right)}{\text{median}\left(\gamma_{x}\left(\alpha \right)\right)}, \end{array}$$

where max(·) is the operator to get the maximum, and median(·) operator is the median of CFSD. Therefore, a high quality signal would have an outstanding peak in CFSD. Consequently, the quality indication would have a high value. An example is given in Figure 5 to show the value of the indicator corresponding to the degree of periodicity. It can be seen that, for an “unacceptable” signal, there is much more random noise than that in the “acceptable” signal. The CFSD of the “unacceptable” signal has no dominant peak, and the indicator has a small value, as shown in Figures 5(a) and 5(b). However, the CFSD of the “acceptable” signal has a dominant peak due to a higher degree of periodicity, shown in Figure 5(c) and 5(d). Hence, the indicator has a larger value.

#### 2.4.5. Summary of the Features

Features used in the work to measure of signal quality are summarized in Table 3. A new frequency-smoothed envelope was proposed in subsection 2.4.2. Therefore, the envelope-related features indexed by “5-9” in Table 3 are novel in signal quality assessment. Degree of periodicity indexed by “10” was an effective feature proposed by the authors' team previously.

### 2.5. SVM-Based Binary Classification

**Figure 6:**
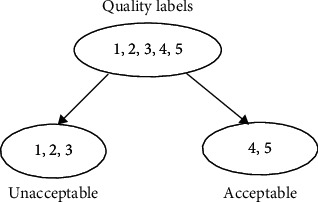
Binary classification for signal quality.

This study tries to perform two types of classification. One is to classify signal quality as “unacceptable” and “acceptable.” The rating labels for “unacceptable” include“1,” “2,” and“3.” Meanwhile, the rating indicators for “acceptable” are “4” and “5.” The scheme for binary classification is shown in Figure 6. This classification is a typical two-category classification problem. The well-known SVM-based two-class model is used for this purpose [36, 37].

### 2.6. SVM-Based Triple Classification

**Figure 7:**
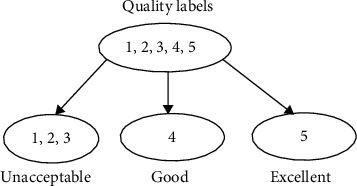
Triple classification for signal quality.

The other type of classification is a triple classification as shown in Figure 7. The signal quality is classified into three classes, i.e., “unacceptable” (quality labels “1”, “2” and “3”), “good” (quality label“4”), and “excellent” (quality label 5). The support vector machine is fundamentally a two-class classifier. Various methods have been proposed for combining multiple two-class SVMs in order to build a multiclass classifier [36]. The “one-versus-one” approach is used here. That is, to train individually three different two-class SVM classifiers on all possible pairs of classes. The first is for “unacceptable” and “good,” ignoring “excellent.” The second is for “unacceptable” and “excellent,” ignoring “good.” The third is for “good” and “excellent,” ignoring “unacceptable.” For each individual classifier, one target label is taken as the positive class and another is taken as the negative class, characterized by a coding matrix. Then, classify a test input according to which class has the highest number of votes. Therefore, a predesigned decoding scheme robust to ambiguity is needed. The study used a simple way to design the decoding scheme based on the number of votes of the submodels' output. For example, if the three submodels outputted {“unacceptable”}, {“unacceptable”}, and {“good”}, respectively, the final decision was {“unacceptable”}, because the number of votes for {“unacceptable”} was greater. However, if the three submodels outputted {“unacceptable”}, {“excellent”}, and {“good”}, respectively, the final decision was manually set as {“unacceptable”} to resolve the ambiguity and avoid producing a possible bad results.

## 3. Results

### 3.1. Performance Indicators for Binary Classification

In the first type of classification, signal quality is classified into two classes, “unacceptable” and “acceptable.” The classification performance is calculated from the number of recordings classified as “unacceptable” or “acceptable” for each of the target classes. The confusion matrix of classification output is like Table 4. Therefore, specificity rate and true positive rate for “unacceptable” and “acceptable” are defined in the next.

(13) $$\begin{array}{c} {SP}_{b\_u}=\frac{N_{uu}}{N_{uu}+N_{ua}},\\ {TP}_{b\_a}=\frac{N_{aa}}{N_{au}+N_{aa}}. \end{array}$$

True negative rate and sensitive rate for “unacceptable” and “acceptable” are

(14) $$\begin{array}{c} {TN}_{b\_u}=\frac{N_{uu}}{N_{uu}+N_{au}},\\ {SE}_{b\_a}=\frac{N_{aa}}{N_{aa}+N_{ua}}. \end{array}$$

The accuracy rate for binary classification is

(15) $$\begin{array}{c} {ACC}_{b}=\frac{N_{uu}+N_{aa}}{N_{uu}+N_{ua}+N_{au}+N_{aa}}. \end{array}$$

It is known from Table 2 that the number of “unacceptable” records is the sum of the number of labels “1,” “2,” and “3,” and a total of 4386; meanwhile, the number of “acceptable” is the sum of the number of labels “4” and “5” and a total of 3507. Therefore, the number of the two classes is an imbalance. A fair overall rate to evaluate the performance of binary classification gives equal weight to the rates defined by (13) and (14)

(16) $$\begin{array}{c} {\text{OR}}_{b}=\frac{{SP}_{b\_u}+{TP}_{b\_a}+{TN}_{b\_u}+{SE}_{b\_a}}{4}. \end{array}$$

### 3.2. Features' Distribution

**Figure 8:**
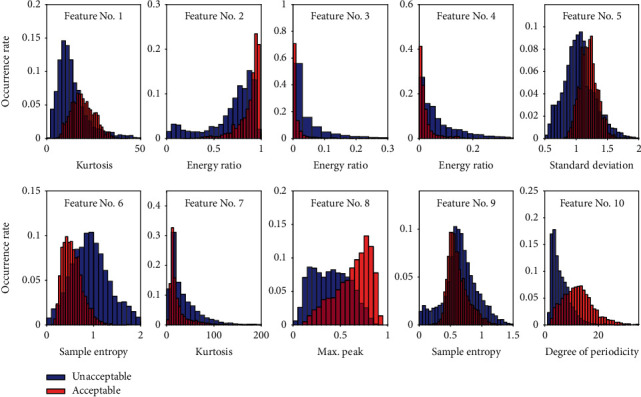
Occurrence rates of the features over binary classification.

**Figure 9:**
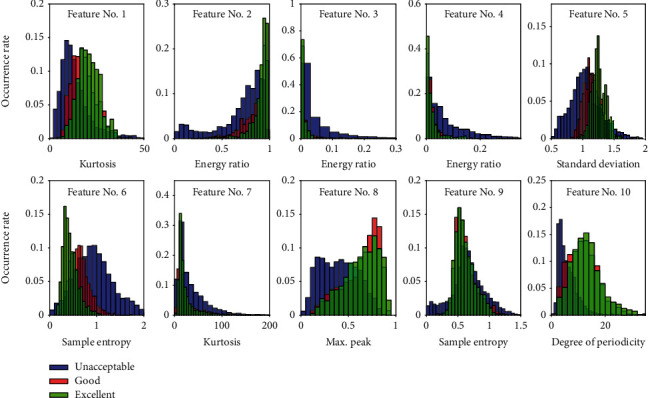
Occurrence rates of the features over Triple classification.

The features extracted from a recording are random variables. They must have difference over quality categories. One possible way to show the difference is to analyze the features' distribution.  Figure 8 gives the occurrence rates of the ten features over “unacceptable” and “acceptable” where the red color is for “acceptable,” and the blue color is for “unacceptable”. The occurrence rate is calculated based on frequency histogram. It is a ratio of number of occurrences in a bin to the total number of occurrences. It is found from visual check that some features have big difference over the two categories, such as the 10^th^ feature (degree of periodicity), the 8^th^ feature (maximum peak in the normalized autocorrelation function), and the 4^th^ feature (energy ratio of high frequency band). However, some features have little difference where the distributions are almost overlapped, such as the 1^st^ feature (kurtosis of heart sound signal), the 2^nd^ feature (energy ratio of low frequency band), and the 9^th^ feature (sample entropy of the autocorrelation function).  Figure 9 gives the occurrence rates over three categories, i.e., “unacceptable,” “good,” and “excellent.” It can be seen that the difference between “good” and “excellent” is much smaller than that between “unacceptable” and “good.” We could conclude that the bigger difference a feature distribution has over the quality categories, the greater contribution the feature could have to discriminate signal quality. Therefore, the performance to discriminate “unacceptable” and “acceptable” must be better than that to classify “unacceptable,” “good,” and “excellent.” The differences in features' distributions prove that the extracted features are effective in quality classification.

### 3.3. Results of Binary Classification

The data is divided randomly in nonoverlap into two categories: training set and test set. To validate the generalization ability of the binary classifier, the ratio of the number of recordings in the training set is 10% and increased by 10% until the rate reaches to 90%. Each test repeats 100 times. The performance is shown in Table 5. It can be seen that the performance indicators slightly increase with the increasing of the percent of data used to train the network. All indicators have low standard deviation. It means that the classifier has stable performance regardless of the training data. Both the accuracy rate and the overall rate reach to 90%, even 10% of the data are used to train. This proves that the classifier has excellent generalization ability from training to testing. As the training data reaches to 90%, both the accuracy rate and overall rate are greater than 94%. On the other hand, the accuracy rate and the overall rate are comparable regardless of the percentage of train data. The recording number for “unacceptable” and “acceptable” is imbalance (One is 4386 and the other is 3507). It seems that imbalance data has little impact on the classification performance.

### 3.4. Performance Indicators for Triple Classification

Similarly, the confusion matrix of the triple classification output is shown in Table 6. Sensitive rates for “unacceptable,” “good,” and “excellent” are defined as

(17) $$\begin{array}{c} {SE}_{t\_u}=\frac{N_{uu}}{N_{uu}+N_{ug}+N_{ue}},\\ {SE}_{t\_g}=\frac{N_{gg}}{N_{gu}+N_{gg}+N_{ge}},\\ {SE}_{t\_e}=\frac{N_{ee}}{N_{eu}+N_{eg}+N_{ee}}. \end{array}$$

Positive predictive rates for “unacceptable,” “good,” and “excellent” are

(18) $$\begin{array}{c} {PP}_{t\_u}=\frac{N_{uu}}{N_{uu}+N_{gu}+N_{eu}},\\ {PP}_{t\_g}=\frac{N_{gg}}{N_{ug}+N_{gg}+N_{eg}},\\ {PP}_{t\_e}=\frac{N_{ee}}{N_{ue}+N_{ge}+N_{ee}}. \end{array}$$

Usually, the accuracy rate is the scale of accurate classified recordings to all recordings

(19) $$\begin{array}{c} {ACC}_{t}=\frac{N_{uu}+N_{gg}+N_{ee}}{N_{uu}+N_{ug}+N_{ue}+N_{gu}+N_{gg}+N_{ge}+N_{eu}+N_{eg}+N_{ee}}. \end{array}$$

Similarly, a fair performance indicator is the overall rate, which is the average of the rates defined by (17) and (18).

(20) $$\begin{array}{c} \frac{{\text{OR}}_{t}={SE}_{t\_u}+{SE}_{t\_g}+{SE}_{t\_e}+{PP}_{t\_u}+{PP}_{t\_g}+{PP}_{t\_e}}{6}. \end{array}$$

### 3.5. Results of Triple Classification

The training scheme for triple classification is the same as that in binary classification. The performance is shown in Table 7. It is seen that *SE*_*t*_*u*_ has the highest score. It means that “unacceptable” recordings are seldom classified as “good” and “excellent” regardless of training conditions. *PP*_*t*_*u*_ has the second highest score. That is to say, the recognized “unacceptable” recordings are seldom from “good” and “excellent.” We may conclude that the classifier has the highest reliable identification for “unacceptable” recordings. *SE*_*t*_*g*_, *SE*_*t*_*e*_, *PP*_*t*_*g*_, and *PP*_*t*_*e*_ have lower score. That is to say, it is hard to classify “excellent” and “good” recordings. In this study, three experts did manual annotation as gold quality labels for each recording. The experts generally had common ideas on the classification of “unacceptable” and “acceptable.” However, they often had different ideas on “good” and “excellent.” Therefore, the manual quality label was somewhat not optimal. The authors believe this was the top reason for the low recognition rate between “good” and “excellent.” It is not a surprise, as even the experts who performed annotations usually have different ideas on a recording to be classified as “good” or “excellent.” It is seen from Table 7 that the overall rate is lower than the accuracy rate. This difference may be caused by the imbalance number (the numbers of “unacceptable,” “good,” and “excellent” are 4386, 1950, and 1557, respectively). Therefore, the heavy imbalance is obvious.

The authors obtain the results (Table 5 and Table 7) using Monte Carlo computer simulations. These results are calculated based on 100 times of random repeat. The numbers are presented in mean ± standard deviation to show the performance stability. It can be seen that the performance increases with the percent of data to train increasing. The standard deviations are generally not greater than 1%. This proves that the classifier has a very stable output even in many times of random repeat.

## 4. Discussions

### 4.1. Analysis of Feature Effectiveness by Sequential Forward Feature Selection

In this study, 10 features are used for the classification of signal quality. It is interesting to know how much effective a feature is in the quality classification. Forward feature selection is an algorithm for this purpose. The selection criteria involve the minimization of the average of the classification errors. A sequential search algorithm which adds or removes features from a candidate subset while evaluating the criterion. Since an exhaustive search of all possible feature combinations is infeasible, the sequential searches will move in only one direction. Sequence forward feature selection is that the feature subset starts from an empty set, and each time one feature is selected to be added to the feature subset, until the feature function is optimal. Generally speaking, every time a feature is selected that makes the value of the evaluation function optimal. Therefore, sequential forward feature selection is a way to evaluate the degree of effectiveness of the features. The sequential order of the selected features for binary classification is given in Table 8. It can be seen that the overall rate increases with increasing trial number. The feature indexed by “10”, i.e., degree of periodicity, gives the highest accuracy of 73.1%, for binary classification. It proves that the degree of periodicity is an efficient indicator for signal quality. The features indexed by “10,” “8,” “4,” “5,” and “3” were the top five features to yield accuracy 92.1%. The other features contribute little to the classification. These results of sequential order revealed by sequential forward feature selection are consisted with those observations in features' probability distribution, shown in Figure 8. We can find that the 10^th^ and 8^th^ features' probability distribution has greater difference over binary classification than the other features. It is not surprise that they rank top two. However, the features indexed by 9, 1, and 6 have less difference over categories. Therefore, they rank bottom. We can see that the envelope-related new features (indexed by 8, 5, 7) have great contribution in signal quality discrimination.

### 4.2. Previous Methods and Performance Comparisons

Previous researchers have proposed several techniques for the assessment of heart sound signal quality [15–18]. Mubarark et al. introduced three types of time domain features for the classification of signal quality [18]. The feature set comprised of root mean square index, zero crossing ratio, and window ratio. One feature is the root mean square of successive differences. If a heart sound recording has high quality and is suitable for further processing, this feature is expected to be less than a threshold. The zero crossing ratio is computed as the ratio of zero crossing number to the recording length. Since a noisy recording has a greater number of zero crossings than a clean recording, if the ratio is greater than 0.3, then it refers to a noisy recording. To calculate the window ratio, a recording is divided into a number of windows and each of length 2200 ms. A window is assigned a score of “1” if the number of peaks within the window is in the range of a specified number. The window ratio is defined as the ratio of the number of windows having a score “1” to the total number of windows. In this paper, the optimal value for the specified number is set as 169 based on the grid search algorithm.

Springer et al. proposed the systemic method to evaluate the signal quality in terms of nine indices [17]. The algorithm was tested on 700 recordings collected from 151 adult individuals. The classification accuracy was 0.822 for mobile phone-based acquired data and 0.865 for electronic stethoscope-based recorded data. The Matlab codes for this method were downloaded from the website [38].

Zabihi et al. also proposed a quality detection method in Physionet/Cinc Challenge 2016 [15]. In the approach, they used 18 types of features from time, frequency, and time-frequency domains without segmentation. These features were fed into an ensemble of 20 feed-forward neural networks for the quality classification task. The code to extract these features is available at Physionet website [39].

**Figure 10:**
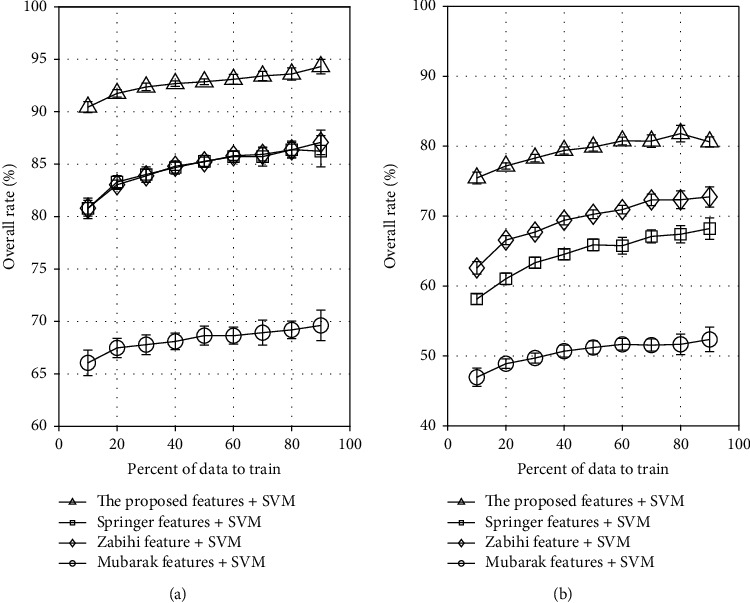
Performance comparison to previous methods. (a) Binary classification. (b) Triple classification.

The performance of our proposed method is compared with three baseline methods [14, 16, 17] and depicted in Figure 10. To show the performance difference of the features proposed by previous research groups, each method was implemented separately by feeding the features to an SVM classifier.  Figure 10(a) shows the performance of a binary classification. The proposed features have the best performance where the overall rate is greater than 0.9 even if 10% of the data are used to train the classifier. Springer's feature and Zabihi's feature have similar performance where their curves are almost overlapped regardless of training percent. The overall rates for baseline methods lie in the range of 81% to 87% in this study. These performances are comparable to those of their studies in their own data.  Figure 10(b) shows the performance of triple classification. It shows that the proposed features give better performance for both binary and triple classification. However, Zabihi's and Springer's features give moderate performance and Mubarak's features give not good enough performance, respectively.

Computer experiments show that Springer's method takes more CPU time than that of the proposed Zabihi's and Mubarak's method because Springer's method involves a heart sound segmentation process, which takes a very large computation load. Moreover, the performance of the segmentation process has an impact on quality assessment.

## 5. Conclusions

This paper has presented a method for the heart sound signal quality assessment. It used ten types of multidomain features to evaluate the heart sound quality through 7893 recordings from the heart sound databases. Experts performed manual annotations for each recording as gold standard quality labels. Even 10% of the data were used to train the model, and the accuracy rate was over 90%. The binary classifier had good generalization ability indeed. The sequential forward feature selection indicated that the top five features dominate the binary classification. Besides, the accuracy rate reached to 85.7% in the triple classification. Signal quality assessment is a necessary preprocessing step in the automatic analysis of heart sound signals. A good quality of the heart sound signal is helpful to obtain reliable analysis results. The proposed method is widely adaptive to comprehensive recordings collected by different devices, in different environments, and in different data lengths. It could serve as a potential candidate in future automatic heart sound signal analysis in clinical applications.

## Figures and Tables

**Table 1:** Labeling scheme for heart sound signal quality annotation.

Quality label	Quality name	Quality description
1	Very bad	No heart sound can be heard. Only noise or only harmonic signal
2	Bad	Mostly noise but some heart sounds can be heard and identified by the human eyes
3	Borderline	Very weak heart sounds but beating rhythms can be recognized, fairly difficult to interpret
4	Good	Heart sounds can be easily heard and interpretable, but some noise presents
5	Excellent	Almost no noise, heart sounds can be clearly heard, identified by visual check, and interpretable with confidence

**Table 2:** Summary of heart sound recordings.

Database	Original number	Range of recording length (s)	Num. excluding those less than 6 s	Num. of very bad quality	Num. of bad quality	Num. of borderline quality	Num. of good quality	Num. of excellent quality
CinCHS	3153	5.3-121.9	3152	196	471	659	948	878
CTHS	412	17.9-71.1	412	0	135	149	62	66
PASCAL	859	0.7-27.8	454	52	24	124	139	115
CDHS	3875	15.0-34.1	3875	71	1557	948	801	498
Sum	8299	5.3-121.9	7893	319	2187	1880	1950	1557

**Table 3:** Summary of features used in this study.

Feature index	Feature description	Feature index	Feature description
1	Kurtosis of heart sound signal	6	Sample entropy of the envelope
2	Energy ratio of low frequency band	7	Kurtosis of the autocorrelation function
3	Energy ratio of middle frequency band	8	Maximum peak in the normalized autocorrelation function
4	Energy ratio of high frequency band	9	Sample entropy of the autocorrelation function
5	Standard deviation of the envelope	10	Degree of periodicity

**Table 4:** Confusion matrix of the binary classification.

		Predicted class	
		Unacceptable	Acceptable
True class	Unacceptable	*N*_*uu*_	*N*_*ua*_
	Acceptable	*N*_*au*_	*N*_*aa*_

**Table 5:** Performance of binary classification.

Percent of data to train (%)	Percent of data to test (%)	Data overlap	*SP*_*b*_*u*_(%)	*TP*_*b*_*a*_(%)	*TN*_*b*_*u*_(%)	*SE*_*b*_*a*_(%)	*ACC*_*b*_(%)	OR_*b*_(%)
10	90	No	94.4 ± 0.7	85.7 ± 1.3	89.3 ± 0.8	92.4 ± 0.8	90.5 ± 0.5	90.4 ± 0.5
20	80	No	94.7 ± 0.7	88.2 ± 1.2	91.1 ± 0.8	93.0 ± 0.8	91.9 ± 0.4	91.7 ± 0.4
30	70	No	94.6 ± 0.5	89.8 ± 1.0	92.2 ± 0.7	92.9 ± 0.5	92.4 ± 0.4	92.4 ± 0.4
40	60	No	95.1 ± 0.4	89.9 ± 0.8	92.6 ± 0.6	93.5 ± 0.5	92.8 ± 0.3	92.7 ± 0.3
50	50	No	94.9 ± 0.4	90.4 ± 0.7	92.6 ± 0.5	93.4 ± 0.4	93.0 ± 0.3	92.9 ± 0.3
60	40	No	95.4 ± 0.3	90.3 ± 1.0	93.6 ± 0.7	94.0 ± 0.4	93.2 ± 0.5	93.1 ± 0.5
70	30	No	95.2 ± 0.5	91.3 ± 1.1	93.3 ± 0.8	93.8 ± 0.6	93.5 ± 0.4	93.4 ± 0.4
80	20	No	95.4 ± 0.8	91.4 ± 1.2	93.4 ± 0.8	94.1 ± 1.0	93.7 ± 0.6	93.6 ± 0.6
90	10	No	96.1 ± 1.0	92.2 ± 1.2	94.0 ± 0.9	94.9 ± 1.2	94.3 ± 0.7	94.3 ± 0.7

**Table 6:** Confusion matrix of the triple classification.

		Predicted class		
		Unacceptable	Good	Excellent
True class	Unacceptable	*N*_*uu*_	*N*_*ug*_	*N*_*ue*_
	Good	*N*_*gu*_	*N*_*gg*_	*N*_*ge*_
	Excellent	*N*_*eu*_	*N*_*eg*_	*N*_*ee*_

**Table 7:** performance of triple classification.

Percent of data to train (%)	Percent of data to test (%)	Overlap	*SE*_*t*_*u*_(%)	*SE*_*t*_*g*_(%)	*SE*_*t*_*e*_(%)	*PP*_*t*_*u*_(%)	*PP*_*t*_*g*_(%)	*PP*_*t*_*e*_(%)	*ACC*_*t*_(%)	OR_*t*_(%)
10	90	No	95.4 ± 0.9	61.7 ± 3.1	65.4 ± 3.3	88.1 ± 1.1	67.6 ± 1.4	74.6 ± 2.0	81.2 ± 0.5	75.8 ± 0.8
20	80	No	95.6 ± 0.5	66.9 ± 1.8	66.4 ± 2.3	89.9 ± 0.5	69.0 ± 1.3	77.3 ± 1.3	82.7 ± 0.3	77.5 ± 0.5
30	70	No	95.8 ± 0.4	67.5 ± 1.6	69.0 ± 2.0	90.5 ± 0.7	71.0 ± 1.3	77.2 ± 1.1	83.6 ± 0.4	78.5 ± 0.5
40	60	No	95.8 ± 0.4	69.8 ± 1.6	69.4 ± 1.7	91.0 ± 0.5	71.7 ± 1.2	78.7 ± 1.1	84.2 ± 0.4	79.4 ± 0.5
50	50	No	95.9 ± 0.4	69.8 ± 1.5	70.5 ± 1.2	91.6 ± 0.6	72.2 ± 1.0	77.7 ± 1.6	84.4 ± 0.4	79.6 ± 0.6
60	40	No	96.2 ± 0.7	71.0 ± 1.6	72.0 ± 2.3	91.6 ± 0.8	73.5 ± 1.4	79.9 ± 1.1	85.2 ± 0.4	80.7 ± 0.6
70	30	No	95.9 ± 0.6	71.2 ± 2.8	71.4 ± 1.4	92.1 ± 0.7	72.6 ± 1.1	78.5 ± 2.1	84.9 ± 0.6	80.3 ± 0.9
80	20	No	95.9 ± 0.6	71.9 ± 2.6	73.3 ± 2.6	92.6 ± 1.1	73.4 ± 1.8	79.2 ± 1.9	85.5 ± 1.0	81.1 ± 1.2
90	10	No	95.9 ± 0.9	72.9 ± 2.0	72.8 ± 2.5	92.3 ± 1.2	74.1 ± 2.0	80.3 ± 1.7	85.7 ± 0.6	81.4 ± 0.7

**Table 8:** Sequential forward feature selection in binary classification.

Trial no.	Sequential order of feature index	Overall rate of 5-fold validation (%)
1	10	73.1
2	10, 8	84.7
3	10, 8, 4	89.8
4	10, 8, 4, 5	91.0
5	10, 8, 4, 5, 3	92.1
6	10, 8, 4, 5, 3, 7	92.5
7	10, 8, 4, 5, 3, 7, 9	93.0
8	10, 8, 4, 5, 3, 7, 9, 2	93.4
9	10, 8, 4, 5, 3, 7, 9, 2, 1	93.7
10	10, 8, 4, 5, 3, 7, 9, 2, 1, 6	94.0

## Data Availability

The heart sound data and manual labels are available at https://pan.baidu.com/s/1PHJO0ZSJds7NDur0CfpG2Q. The password is for the access is nk17. The matlab codes for this study are shared at https://github.com/tanghongdlut/signal-quality-assessment-of-heart-sound-signal.

## Abbreviations

STFT: Discrete short-time Fourier transform
CFSD: Cycle frequency spectral density
SVM: Support vector machine.

## References

[1] Sakamoto T., Kusukawa R., Maccanon D. M., Luisada A. A. (1965). Hemodynamic determinants of the amplitude of the first heart sound. *Circulation Research*.

[2] Luisada A. A., Liu C. K., Aravanis C., Testelli M., Morris J. (1958). On the mechanism of production of the heart sounds. *American Heart Journal*.

[3] Erickson B. (2009). *Heart sounds and murmurs across the lifespan (fourth edition)*.

[4] Dwivedi A. K., Imtiaz S. A., Rodriguez-Villegas E. (2019). Algorithms for automatic analysis and classification of heart sounds – a systematic review. *IEEE Access*.

[5] Clifford G. D., Liu C., Moody B. (2017). Recent advances in heart sound analysis. *Physiological Measurement*.

[6] Semmlow J. L. (2016). Improved heart sound detection and signal-to-noise estimation using a low-mass sensor. *IEEE Transactions on Biomedical Engineering*.

[7] Roy J. K., Roy T. S., Mukhopadhyay S. C. (2019). Heart sound: detection and analytical approach towards diseases, modern sensing technologies. *Spring*.

[8] Beritelli F., Spadaccini A. Heart sounds quality analysis for automatic cardiac biometry applications.

[9] Tang H., Li T., Park Y., Qiu T. (2010). Separation of heart sound signal from noise in joint cycle frequency-time-frequency domains based on fuzzy detection. *IEEE Transactions on Biomedical Engineering*.

[10] Tang H., Zhang J., Sun J., Qiu T., Park Y. (2016). Phonocardiogram signal compression using sound repetition and vector quantization. *Computers in Biology and Medicine*.

[11] Li T., Tang H., Qiu T. (2011). Best subsequence selection of heart sound recording based on degree of sound periodicity. *Electronics Letters*.

[12] Li T., Tang H., Qiu T. (2013). Optimum heart sound signal selection based on the cyclostationary property. *Computers in Biology and Medicine*.

[13] Abdollahpur M., Ghaffari A., Ghiasi S., Mollakazemi M. J. (2017). Detection of pathological heart sounds. *Physiological Measurement*.

[14] Naseri H., Homaeinezhad M. R. (2012). Computerized quality assessment of phonocardiogram signal measurement-acquisition parameters. *Journal of Medical Engineering & Technology*.

[15] Zabihi M., Rad A. B. (2016). Heart sound abnormaly and quality detection using ensemble of neural networks without segmentation. *Computing in Cardiology*.

[16] Springer D. B., Brennan T., Zuhlke L. J. Signal quality classification of mobile phone-recorded phonocardiogram signals.

[17] Springer D. B., Brennan T., Ntusi N. (2016). Automated signal quality assessment of mobile phone-recorded heart sound signals. *Journal of Medical Engineering & Technology*.

[18] Mubarak Q., Akram M. U., Shaukat A. A., Hussain F., Khawaja S. G., Butt W. H. (2018). Analysis of PCG signals using quality assessment and homomorphic filters for localization and classification of heart sounds. *Computer Methods and Programs in Biomedicine*.

[19] Liu C., Springer D., Li Q. (2016). An open access database for the evaluation of heart sound algorithms. *Physiological Measurement*.

[20] May 16, 2019, https://www.physionet.org/physiobank/database/challenge/2016/

[21] May 16, 2019, http://www.peterjbentley.com/heartchallenge/

[22] May 16, 2019, http://www.diit.unict.it/hsct11

[23] Spadaccini A., Beritelli F. Performance evaluation of heart sounds biometric systems on an open dataset.

[24] Rekanos I., Hadjileontiadis L. (2006). An iterative kurtosis-based technique for the detection of nonstationary bioacoustic signals. *Signal Processing*.

[25] Saragiotis C., Hadjileontiadis L., Rekanos I., Panas S. (2004). Automatic P phase picking using maximum kurtosis and&lt;tex&gt;$kappa$&lt;/tex&gt;-Statistics criteria. *IEEE Geoscience and Remote Sensing Letters*.

[26] Cain Meghan K., Zhang Z., Yuan K. H. (2017). Univariate and multivariate skewness and kurtosis for measuring nonnormality: prevalence, unfluence and estimation. *Behavior Research Methods*.

[27] Decarlo L. T. (1997). On the meaning and use of kurtosis. *Psychological Methods*.

[28] Choi S., Jiang Z. (2008). Comparison of envelope extraction algorithms for cardiac sound signal segmentation. *Expert System with Applications*.

[29] Liang H., Lukkarinen S., Hartime I. (1997). Heart sound segmentation algorithm based on heart sound envelogram. *Computers in Cardiology*.

[30] Jiang Z., Choi S. (2006). A cardiac sound characteristic waveform method for in-home heart disorder monitoring with electric stethoscope. *Expert Systems with Applications*.

[31] Gupta C. N., Palaniappan R., Swaminathan S., Krishnan S. M. (2007). Neural network classification of homomorphic segmented heart sounds. *Applied Soft Computing*.

[32] Richman J. S., Moorman J. R. (2000). Physiological time-series analysis using approximate entropy and sample entropy. *American Journal of Physiology. Heart and Circulatory Physiology*.

[33] Yuenyong S., Nishihara A., Kongprawechnon W., Tungpimolrut K. (2011). A framework for automatic heart sound analysis without segmentation. *Biomedical Engineering Online*.

[34] Jose A. D., Collison D. (1970). The normal range and determinants of the intrinsic heart rate in man. *Cardiovascular Research*.

[35] Kumar D., Carvalho P., Antunes M., Paiva R. P., Henriques J. (2011). Noise detection during heart sound recording using periodicity signatures. *Physiological Measurement*.

[36] Bishop C. M. (2006). *Pattern Recognition and Machine Learning*.

[37] Tang H., Dai Z., Jiang Y., Li T., Liu C. (2018). PCG Classification Using Multidomain Features and SVM Classifier. *BioMed Research International*.

[38] May 16, 2019, https://github.com/davidspringer

[39] August 13, 2019, https://alpha.physionet.org/content/challenge-2016/1.0.0/

